# Disparities in Influenza Control and Surveillance in Latin America and the Caribbean

**DOI:** 10.3390/v17020225

**Published:** 2025-02-05

**Authors:** Tatiana Hoyos-Cerón, Froylán Albarrán-Tamayo, Bernardo Bañuelos-Hernández, María Aurora Londoño-Avendaño

**Affiliations:** 1 Department of Microbiology, College of Health, Universidad del Valle, Calle 4B # 36-00 Ed. 120, Cali 760043, Colombia; tatiana.hoyos@correounivalle.edu.co; 2 Facultad de Veterinaria, Universidad de la Salle Bajío, Avenida Universidad 602, Lomas del Campestre, Leon 37150, Guanajuato, Mexico; falbarrant@lasallebajio.edu.mx

**Keywords:** influenzavirus, vertebrates, incidence, typing, control, surveillance, One Health

## Abstract

To identify measures that mitigate the impact of influenza in Latin America and the Caribbean, we compared the burden and detection capacity in humans and animals after the 2009 pandemic. The incidence rate in people was higher in Chile (23.72 per 100,000 people), but the impact was greater for Guatemala (503.78 disability-adjusted life years per 100,000 people). Brazil, Peru, Argentina, and Mexico built better medical testing, with typing being less frequent in Chile and Argentina, where costs for medical care were higher. The positivity rate among avian and nonhuman mammals was 5.8%, with more cases in Mexico, but constant testing in Chile. H5N1, H5N2, and H7N6 are deadly to poultry, whereas H1N1 is common in swine, and H3N8 in equines. By June 2023, H5N1 had caused severe influenza in two persons and killed millions of birds and hundreds of mammals with aquatic lifestyles. An analysis of the efforts in response to this outbreak revealed that handling of outbreaks in animals needs homogeneity and reinforcement of vaccination. Surveillance in exposed individuals requires articulation of medical and animal health authorities, and the region also demands decentralized typing and networks for genomic characterization.

## 1. Introduction

Influenza is an acute viral infection of avians and mammals caused by enveloped viruses with multisegmented ssRNA(−) genomes of the family Orthomyxoviridae [1,2]. This family includes the species *Alphainfluenzavirus influenzae*, *Betainfluenzavirus influenzae*, *Gammainfluenzavirus influenzae*, and *Deltainfluenzavirus influenzae*, referred to hereinafter as AIV, BIV, GIV, and DIV, respectively [1,3]. AIV can cause seasonal epidemics and pandemics in humans, poultry, cattle, and their wild relatives. BIV and GIV are naturally restricted to humans and swine, but BIV has important morbidity in children and elderly persons, and GIV is rare and causes mild infections [4,5]. DIV is an emerging pathogen of swine and cattle whose capacity to infect humans remains controversial [6,7,8].

There are 18 recognized variants of the haemagglutinin protein (H) in AIV that are determinants of virulence and tropism; these, combined with any of the 11 variations of the glycoprotein neuraminidase (N), constitute the subtypes of this species [1,9]. BIV subdivides in the Victoria and Yamagata lineages have fewer antigenic changes and a mutation rate estimated at 0.5 × 10^−3^ mutations/site/year, five times lower than that of AIV (2.6 × 10^−3^ mutations/site/year) [5,10,11]. AIV subtypes that infect birds, categorized as low pathogenic and highly pathogenic avian influenza viruses (LPAI and HPAI, respectively), can glycosylate sialic acid molecules of vertebrates consisting of α2-3 linkages [12,13]. These patterns are particularly abundant in the respiratory and gastrointestinal tracts of waterfowl, the main reservoirs of the H5N1, H7N7, and H9N2 lineages, while AIV isolates from humans preferentially bind sialic acid with α2-6 linkages; moreover, in humans, α2-3 glycosylation is restricted to cells in the inferior respiratory tract, which makes avian flu uncommon in people unless exposure to a high inoculum has occurred. HPAI lineages are more prone to proteolytic activity of the host, facilitating viral fusion, a crucial step in the replicative cycle of the virus [14,15,16].

Subtypes of AIV H1N1, H1N2, and H3N2, which were originally circulating in swine, have evolved through the generation of amino acids with more affinity to the glycosylation pattern predominant in the human upper respiratory tract. Genotypes of “swine-flu” associated with pandemics have experienced exchanges of genetic segments plus mutations that increase fitness and transmissibility, putting haemagglutinin in an antigenic context to which the human population has never been exposed [5]. Additionally, influenza viruses harbouring neuraminidase with a greater capacity to bind calcium and those whose haemagglutinin covers a greater surface are more tolerant to low pH and low temperatures; these types of variants are more efficient in oral–faecal transmission to waterfowl [17,18] and facilitate contamination among farms through food supplies [19,20].

Orthomyxoviruses mainly affect the upper respiratory tract and cause self-limited infections, with up 50% of the infections being asymptomatic [21]. Humans, poultry, and cattle can develop mild to severe AIV infections depending on the virus–receptor affinity, proteolytic capability, and replicative efficiency given the evolutionary background of the infecting subtype. Transmission among individuals occurs through respiratory aerosols or droplets emitted by exhalation, coughing, or sneezing [22]; animal-to-human contagion can also occur through contact with excreta and respiratory secretions of infected animals [23]. Hence, the highest risk of zoonotic transmission of AIV is between workers in the poultry and swine industries. Over the last 100 years, four AIV pandemics have occurred: the Spanish flu in 1918–1919, the Asian flu in 1957–1958, the Hong Kong flu in 1976–1968, and the swine flu (H1N1pdm09) in 2009. The latter is believed to have originated from pig farms in Mexico.

Zoonotic outbreaks of AIV in Latin America and the Caribbean (LAC) have been reported to occur at significantly lower frequencies than in other continents. Actions to overcome the threat of pandemic influenza were taken after 2009, and human cases are believed to have diminished in 2020–2021 due to biosafety measures during the COVID-19 pandemic; however, as many countries in the region prioritized this emergency, surveillance and vaccination for influenza were left unattended. Moreover, in the first six months of 2023, twenty-one registries of H5N1 in animals were deposited in the repository of the World Organization for Animal Health System (WAHIS); symptomatic infection was confirmed in nearly one million specimens, including aquatic mammals, poultry and waterfowl. The H5N1 outbreak, which started in October 2022, has increased the records of avian flu in LAC by nearly 160 times compared to the annual records for 2016–2019; understanding its impact has great relevance, because it has also caused symptomatic infection in people in contact with wild birds, and more recently with dairy cows [24].

This work is a descriptive analysis of data on influenza collected from 1 January 2009 to 31 June 2023. The exploitative economic model, limited access to testing technologies, and the complexities of the ecosystems where reservoirs can establish make LAC a fertile environment for the appearance of genotypes with pandemic potential. Previous publications on the epidemiology and burden of influenza in this region, summarized in [25,26,27,28,29], are focused on either human health or animal flu; to fill in this gap, we used unified criteria to compare the impact, the detection capacity, and response to outbreaks in these countries to reveal key aspects that reinforce influenza surveillance in the region and provide insights for the integration of public health policies with productive models, ecosystem services, and agricultural legislation.

## 2. Materials and Methods

### 2.1. Approach and Data Sources

We chose for comparison the impact of the disease, trends in screening/typing, and response to outbreaks. Information was extracted from databases containing multicountry records of cases of influenza, or other variables relevant for their interpretation, from 1 January 2009 to 31 June 2023; however, some databases did not contain updated information as of that date. Table 1 lists these resources and indicates the type of data retrieved from each of them. The impact on human health was evaluated in terms of incidence (age standardized cases per 100,000, as calculated in FluNet), disability-adjusted life years (DALYs, as calculated by Global Burden of Disease), and direct economic cost (estimated from Alvis-Guzmán et al. 2018 [30] by adjusting the percentage of inflation). For the impact on animal health, the number of positive animals, animals at risk, and dead animals, either due to having the disease or having been sacrificed, were calculated with data from the Influenza Research Database (IRD) [31] and WAHIS; the economic impact in agriculture was explored in terms of the value in US dollars of meat exports (records from FAO).

Screening trends were determined as counts of human (FluNet data) or animal samples (IRD + WAHIS data) processed per country per year, and put in the context of population growth or animal production to show relationships between public health policies and viral epidemiology; data on typing (genotypes reported per country per year) are shown to indicate installed capacity in influenza surveillance.

The response to outbreaks was evaluated only for animal health, with information from recent H5N1 events in LAC reported in WAHIS. Scoring included 33 possible actions that can be taken. Six surveillance actions were scored as 0–1 (performed = 1, not performed = 0); twenty-four control actions were scored as 0–1 (performed = 1, not performed = 0); the complementary actions, i.e., flu vaccine availability for animals, were scored as 1–2 (not available = 0, available = 1, H5N1 available = 2); and the others were scored as performed = 1, not performed = 0. The maximum score per an event was 34. Countries in whose events a greater number of surveillance and control actions were taken had better response scores, but a penalty was assigned if at least one HPAI event had occurred in the country between 2010 and October 2022 (scored yes = −10, no =−1).

### 2.2. Processing and Exclusion Criteria

The original data were filtered to include only Latin American and Caribbean independent countries, whereas American colonial territories were grouped together as British, French, or other territories, and used as reference values when applicable. No LAC country was totally excluded; however, in analyses where data from different sources were combined, those countries with no records for one of the variables were keep out. All analyses were performed in R version 3.6.1 using the package tidyverse for calculations and ggplot2 and heatmaply for figure creation. The final, combined, datasets are available upon request. Calculations included: (i) adding of age standardized cases per 100,000 per year and country; (ii) adding of DALYs per year and country; (iii) adding the annual percentage of inflation to direct economic cost; (iv) adding the number of animals tested and positives, as well as at risk and dead animals per year, country, and serotype; and (v) adding of poultry and swine heads and meat exports per country and year. Results are reported in accordance with recommendations from the guidelines for accurate and transparent reporting of health estimates (GATHER) [33], guidelines of meta-epidemiological methodology research [34], and guidelines for reporting One Health epidemiological evidence [35].

## 3. Results

### 3.1. Burdens of Influenza

#### 3.1.1. Burdens of Influenza in LAC Medical Systems

**Figure 1:**
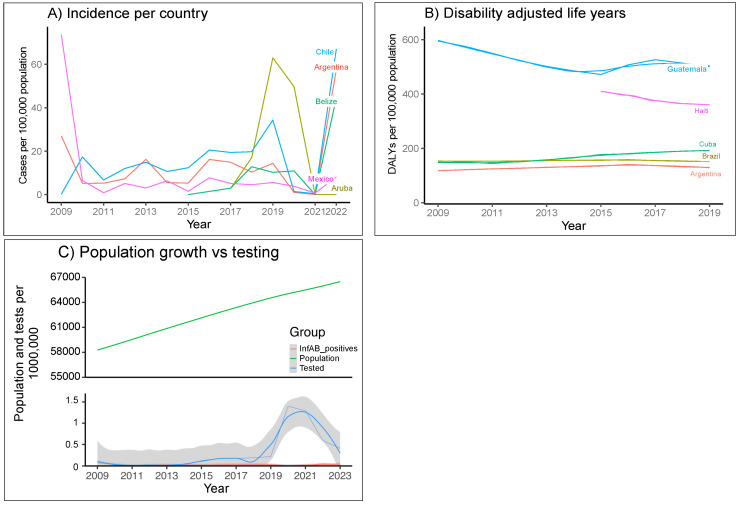
Burden of influenza in medical systems of Latin America and the Caribbean. (**A**) Incidence (age-standardized annual cases of influenza per 100,000 people, all sexes). (**B**) DALYs, based on data from GBD 2020. (**C**) Trends in influenza tests performed in LAC health services, as compared to population growth; curves are plotted with a 90% confidence interval, combining demographic data from the World Bank Databank and epidemiological data from FluNet; values for the 2023 population were generated using an estimated growth of 2.4%.

Incidence of influenza in people, measured as reports of influenza-like illness (ILI), has been estimated to range between 9.9 and 137 cases per 100,000, depending on the sub-region [25,26]. Data of the incidence calculated in this study are summarized in Figure 1A. The average number of human cases per year between 2009 and mid-2023 was 4.86/100.000 people (CI95 2.82–6.91/100,000). Chile and Argentina are among the countries with the most cases of influenza in the last five years (67.02 and 57.50 × 100,000 in 2022, respectively); Caribbean countries refer to less information, except for Aruba (and Belize), where reporting has increased, and a wave in transmission of influenza in Aruba occurred during 2019–2020 (Figure 1A).

When we measured the burden of this disease as disability-adjusted life years (DALYs), Brazil exhibited the highest values, followed by Mexico and Guatemala. Since Brazil and Mexico are the most populated countries in LAC, after these values were adjusted to DALYs per 100,000 people, Guatemala experienced the greatest impact, with an average of 503.78 DALYs × 100,000 (CI95 486–522/100,000) in 2015–2019, followed by Haiti and Cuba (averages of 381.08 and 184.71 DALYs per 100,000, respectively, for the same period) (Figure 1B); Brazil moved down to fourth place with 154.67 DALYs, near Suriname, Colombia, and Uruguay (123.89, 111.14, and 105.28 DALYs × 100,000, respectively), but for Mexico, this indicator was much lower.

In 2011, the direct cost of medical care for a confirmed case of influenza established by Alvis-Guzmán et al. in 2018 ranged between 315 and 4867 Unites States dollars (USD) depending on the country (Supplementary Table S1) [30]. By adjusting the percentage of inflation, we estimated the cost to be 3185 USD on average in 2022 (CI95 1646–4724 USD); medical care was more expensive in Chile, but the most significant increases were observed in Argentina (Supplementary Table S1). Overall, based in this estimation, the economic cost of medical care for influenza in Latin America has doubled since 2011 [30].

Screening is not directly proportional to population growth, but rather is influenced by changes in national public health policies. For example, in most countries, testing tripled during the COVID-19 pandemic, but since 2015, an increase has been observed in the number of individuals who undergo a confirmatory influenza test. Since 2014, screening for influenza has drastically moved from mainly positive cases loaded to databases to a low frequency of positive cases among the samples tested (Figure 1C). In those years, many countries have implemented the sentinel system for the surveillance of respiratory diseases [36], capturing and storing data for ILI and other acute respiratory infections (ARIs). Accordingly, since 2015, the percentage of influenza-positive individuals among respiratory infections has ranged between 0.25% and 19.4% per year, with an average of 10.1% (95% CI 6.8–13.6%) of respiratory samples from symptomatic individuals being AIV- or BIV-positive, as previously reported [27].

#### 3.1.2. Burdens of Influenza in LAC Production Systems

**Figure 2:**
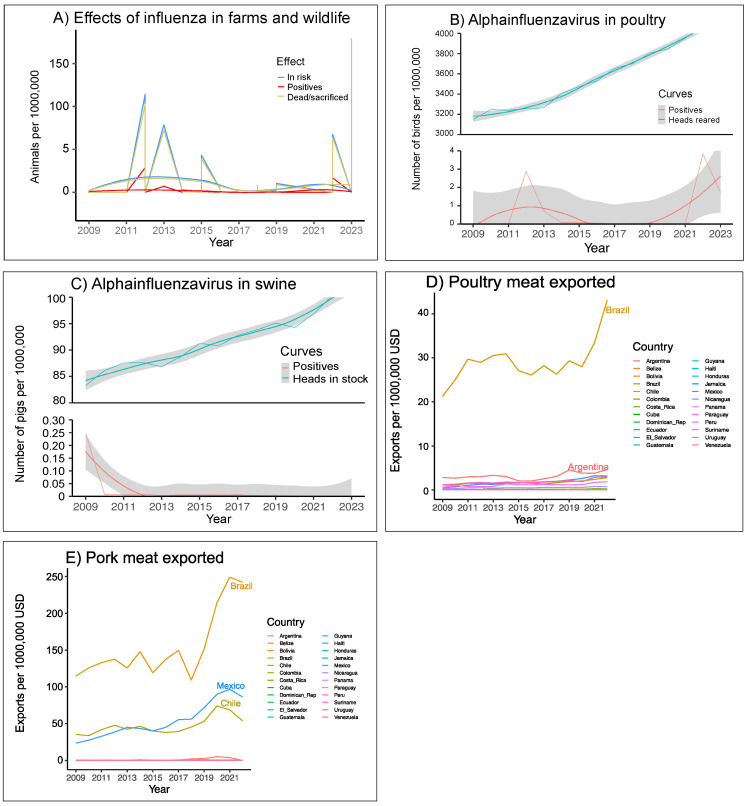
Burden of influenza in animals. (**A**) Effect of influenza in animals. The figures include data for poultry and wild birds (multiple species), swine, equines, and other mammals; positives are cases confirmed reported in both the IRD and WAHIS, but animals at risk and sacrificed correspond to those reported in WAHIS. For (**B**,**C**), produce data were obtained from FAO and AIV cases; both plots combine data from IRD and WAHIS, adjusted by excluding nonpoultry avian hosts and wild boars. For (**A**–**C**), the databases were retrieved during July 2023 and data are plotted with a 90% confidence interval. In (**D**,**E**), we added exports of raw, frozen, and cuts of pig and poultry (chicken, geese, turkey) conducted by countries, based on records from FAO.

The global impact of influenza in animals in LAC can be appreciated in terms of the thousands of animals slaughtered to contain outbreaks in 2012, 2013, 2015, and 2022–2023 by having near contact with positives confirmed (Figure 2A). Although most of the sacrificed were poultry, these data show that equines, swines, and multiple specimens of avian wildlife were submitted to the same measure.

Testing for influenza in the region covers a low percentage of animals in the poultry and swine industries (Figure 2B,C), albeit we estimated the productivity to have increased between 16% and 25% since 2009. The peaks of increase in AIV positivity shown in Figure 2B correspond to outbreaks of H7N3 (Mexico, 2012–2013) and the recent spillover of HPAI H5N1 in birds in 2022–2023; for Figure 2C, the maximum corresponds to the H1N1 epidemic (2009–2010). An average of 0.016% and 0.0004% of poultry and swine heads were tested per year, respectively, with better proportions reached after pandemics (2011, 2022) (details in Supplementary Figure S1A–C). There were deaths of pigs associated with H1N1pdm09 in Brazil and Mexico in 2009 [37,38,39,40]; however, records of dead and slaughtered animals in WAHIS began in 2012, as appreciated in Figure 2A.

From an economic point of view, the exports of poultry meat had minor variations in regard to influenza. In Brazil, this market shows ups and downs that were not clearly linked to the HPAI outbreaks (Figure 2D). The main impact of the H1N1pmd09 outbreak was a decrease in pork exports in Chile plus a slowdown in the industry in the other main exporting countries, which are Mexico and Brazil (Figure 2E); however, Brazil shows a turning point starting in 2018 that has catapulted it as a world leader in exports of pork, poultry, and beef.

### 3.2. Screening and Typing of Influenzaviruses Within LAC

#### 3.2.1. Screening and Typing Within Medical Networks

**Figure 3:**
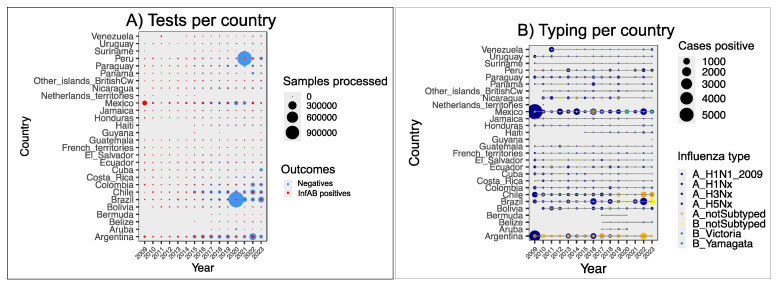
Testing and typing for human influenza in Latin America and the Caribbean. (**A**) Influenza tests performed in LAC each year, according to FluNet data. (**B**) Typing of positive tests in LAC (data from FluNet) are plotted as the number of cases of each influenza genotype per year in each country. We emphasize H serotypes because typing for neuraminidase is less often performed; therefore, the label Nx ranges from not typed at all to multiple N serogroups determined in that year for that country.

The sentinel system for respiratory viruses in the human population has the advantage of reflecting situations additional to the incidence of influenza. As an example, reduced or lack of continuity in testing is observed in Figure 3A,B for several countries, including those experiencing political instability, such as Venezuela and Haiti. A gap in reports for 2021 in countries such as Cuba and Panama is a strong indicator of the impact that the SARS-CoV-2 pandemic has had on influenza surveillance in the region (Figure 3A). Brazil, Peru, Argentina, and Mexico are the countries with the greatest capacity for testing; other countries are either stuck in their capability or have continued reporting the same amount of data since 2015, despite having faced the challenges of providing medical attention and feeding epidemiological data of SARS-CoV-2 during the pandemic.

Surveillance confirmed cases of AIV subtypes H1, H3, and H5 (Figure 3B) but did not fully track their origin to communitarian, nosocomial, and zoonotic transmission. Paraguay, Nicaragua, Argentina, and Brazil are the countries with the lowest proportion of positive tests being genotyped. The two main genotypes of BIV are often detected; however, confirmation and uploading in the notification system are dependent on economics and political stability. Surprisingly, for the first half of 2023, cases of influenza in the region were predominantly caused by BIV.

#### 3.2.2. Screening and Typing in Farms and Wildlife

**Figure 4:**
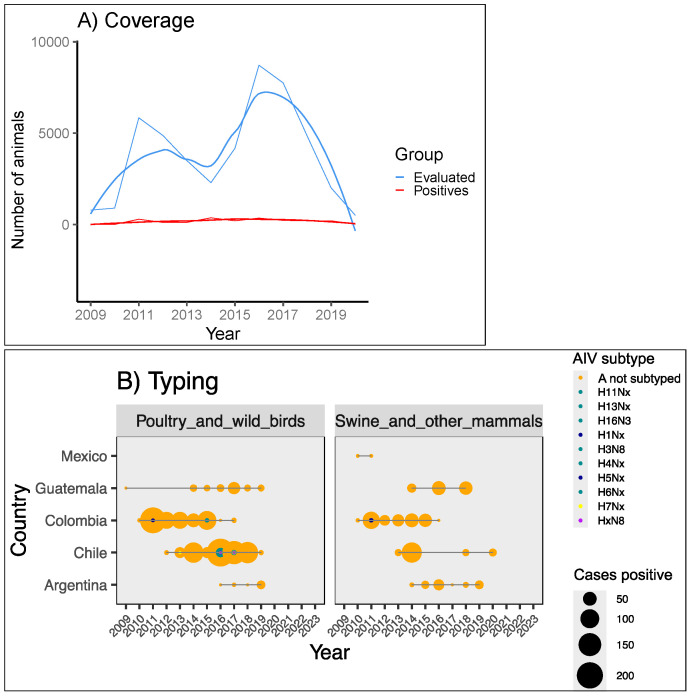
Environmental surveillance for AIV in LAC. (**A**) Coverage in avian and nonhuman mammals, estimated with data from the IRD. (**B**) Typing for samples in environmental surveillance, grouped by avian and mammal hosts. The data were retrieved during July 2023 and were plotted with a 90% confidence interval (Panel A).

Surveillance of influenza among animals is performed through two independent strategies. The first, environmental sampling, is performed to determine influenza circulation in avian and nonhuman mammals; this sampling was found to be carried out mainly by academic institutions, based on the record of the institutions that generate and upload the data in IRD, and characterized by a lack of continuity. Figure 4A shows an increase in environmental surveillance during 2016–2018. However, late-pandemic and post-pandemic data are not being fed into the database for comparison; intermittent patterns of screening, which may be linked to less research and a decrease in budgets, can be appreciated in Figure 4B. Most LAC countries do not report data on influenza occurrence through this strategy in international networks, with Chile and Colombia keeping the longest records of influenza in avian and nonhuman mammals from environmental screening. The average percentage of positives among tested wild avian and nonhuman mammals estimated in this study was 5.79% (CI95 3.78–7.81%); however, in some localities, the percentage of positives reached 24%, similar to the 12% and 37% for poultry and swine, respectively, reported in some studies [41,42].

**Figure 5:**
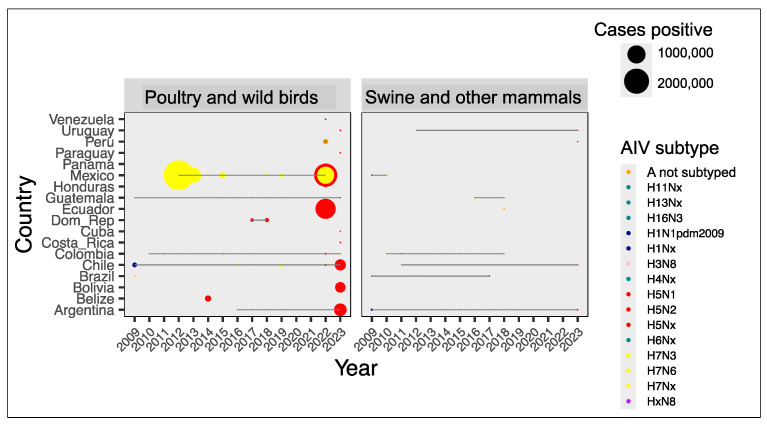
Types of alphainfluenzavirus found in productive systems of Latin America and the Caribbean. These data are typing for influenza in poultry, horses, and swine farms and their neighbouring wild animals, conducted during influenza outbreaks and reported in the WAHIS database.

The second strategy, performed by animal health authorities, focuses on events where at least one symptomatic individual is confirmed to be AIV positive; in this case, the authorities may further identify animals at risk of being infected as part of the measures for disease containment; in many cases, animals at risk are sacrificed (Figure 2A) for further carcass disposition or commercialization as detected in a few registries of WAHIS. The genotypes H5N1, H5N2, and H7N3 (Figure 5) are the most frequently reported in avian hosts, but their host range includes non-avian species, as described in sections below.

One increase in the amount of infections, as shown in the first panel of Figure 5, corresponds to the H7N3 outbreak in Mexico (2011–2012); the second increase, in 2017, was caused by the same genotype in Chile; and the third is the ongoing H5N1 outbreak, as mentioned above. In 2014, the first cases with genotype H5 were detected in the area; ~83,000 moderately ill birds were sacrificed in Belize, and the virus (H5N2) was labelled LPAI. An additional outbreak caused by H3N8 in equids during 2018 is shown in Supplementary Figure S2, where an amplified version of the right box was created by excluding H5Nx data to highlight the tendencies of other AIV genotypes in swine and other mammals. In swine, H1N1pm2009 was notoriously identified in Argentina, Brazil, and Mexico, but types H1N2 and H3N2 were also detected in symptomatic individuals (Supplementary Figure S2); here, we estimated the regional positivity of AIV in swine to be 45% (3643 positives out of 8169 individual pigs and wild boars tested between 2009 and mid-2023, if combining IRD and WAHIS data).

### 3.3. The Menace of HPAI Viruses

By June 2023, the H5N1 genotype had caused the death of more than two million wild and produce birds in Latin America and the Caribbean, and had led to the sacrifice of nearly thirty million animals to contain the outbreaks. Susceptible hosts include multiple bird families (Table 2), but its pathogenic capability extends beyond being an HPAI virus. Lethal infection occurs in at least four families of mammals with different aquatic habitats: Otariidae (i.e., sea lions), Mustellidae (i.e., sea and river otters), Procyionidae (coatis), and Delphinidae (dolphins) (Table 2). Additionally, at the time of data collection, there were two records of H5N1 cases in people in the region.

Only two cases of H5N2 in humans were recorded in FluNet by mid-2023, but this is the third AIV genotype in circulation that is deadly for poultry in LAC. H5N2 was detected in whistling ducks (*Dendrocygna* spp.) in Colombia and Chile, but its pathogenicity in animal populations native to the Americas has not been established. H7 variants have not been detected in the human population in the Americas. The pathogenicity of H7N3 in poultry is comparable to that of H5N1, but less damage has been reported in other birds or waterfowl reservoirs identified in Latin America, such as yellow-billed ducks (*Anas georgica* and *A. flavirostris*). Although H7N9 seems to be absent in Latin America and the Caribbean, it is important to note the number of animals with severe symptoms (deaths) who were not subjected to genotyping (Table 2).

### 3.4. Strengths and Weaknesses in the LAC Influenza Control and Surveillance System for Animal Health

**Figure 6:**
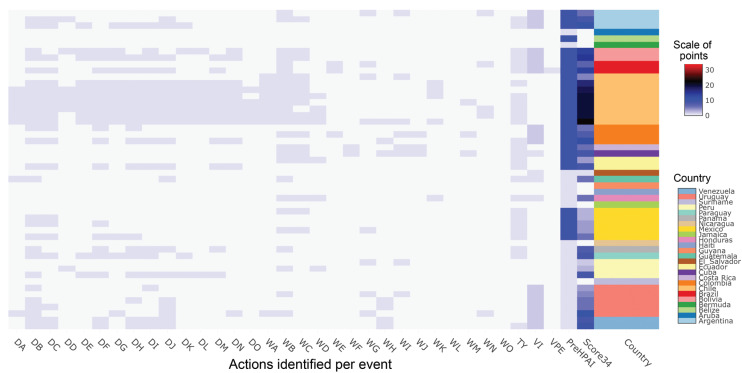
Surveillance, control, and complementary efforts in response to H5N1 events 2022–2023. The scale of points applies for columns DA to Score34; that is, the sum of values assigned in DA to VEP minus PreHPAI. Notice that each row is an event; events are grouped by country, which appear in alphabetical order and in relation to a colour key. Surveillance actions: DA-DC in domestic, and WA-WC in wild birds, where A = surveillance in wildlife reservoirs, B = surveillance within the restricted zone, C = surveillance outside the restricted zone; Control actions: DD-DO in domestic and WD-WO in wild birds, where D = where selective killing and disposal, E = official destruction of animal products, F = quarantine, G = official disposal of carcasses, by-products, and waste, H = stamping out, I = disinfection, J = movement control, K = zoning, L = inactivation in products or by-products, M = traceability, N = screening, O = ante and post-mortem inspections; Complementary actions: TY = typing, VI = vaccines for animals, VPE = vaccination post-event, PreHPAI = HPAI reports previous to H5N1.

Surveillance is characterized by greater efficiency in poultry than in swine farming systems (Figure 4B, Figure 5 and Figure 6). The measures most frequently taken when animal health authorities confirm H5N1 cases in farms are random sampling in areas close to the focus (range of 5 km) and in a broader area (7–10 km), quarantine, and restriction of movement for workers and material (Figure 6). Random sampling does not always include poultry and wild birds and is not supported by in situ typing tools; this would generate new fences only if symptomatic cases are evident, without reducing dispersion through reservoirs. The sacrifice of animals is always recommended; however, this activity was carried out by authorities only in 15 out of the 34 events analysed here. In some HPAI events, prior to H5N1, the carcasses were used as a protein source with no clear regulation for its transportation and handling.

Previous experience with HPAI events had no effect on whether LAC countries are more efficient in their surveillance strategies. Figure 6 shows a score of efficiency by event that reaches 34 points if all surveillance actions among wild and domesticated birds are performed and the country has AIV vaccines. The values indicate that most countries do not apply the same protocol at each event because guidelines are issued locally or are fragmentary. Overall, Chile is the country with the most homogeneous handling of H5N1 events; however, approaching of outbreaks in wild mammals needs to converge into the same modus operandi used in farms of this country.

By mid-2023, most LAC countries reported molecular confirmation of influenza in animals by RT-PCR strategies developed for typing H1N1pdm09 more than a decade ago. The same technique is used in national reference laboratories, with approximately 30% of them reporting partial genotyping results of a simplex RT-PCR that detects only the H5 fragment. Verification by sequencing has been performed in 46% of the cases, but no LAC countries have an active program for genomic surveillance (Supplementary Tables S3 and S4). Although sixty percent (13/23) of LAC actively feeding data into the GISAID have technologies for genomic surveillance, only two of them have more than one institution completing wet lab and bioinformatics procedures (Supplementary Table S4). The vaccination of animals is practically absent, although 12 out of the 25 countries with H5N1 have authorized the use of some type of influenza vaccine for agriculture (details available in Supplementary Table S2); the use of vaccines is rare because of efficacy issues and the lack of national vaccination plans, which leaves immunization as a responsibility of the farmers.

## 4. Discussion

The average number of influenza cases per year in LAC over the study period was 30,193 (CI95 18,097–42,290), showing a slight decrease compared with the estimated 36,080 per year between 1980 and 2008 [27]. Several discrepancies between the burden and incidence of human influenza were found; Chile, Aruba, and Argentina had the highest incidence in the last five years (averages of 23.72, 22.08 and 16.38 per 100,000, respectively), but Guatemala, Haiti, and Cuba experienced the greatest impacts, with averages of 503.78, 381.08 and 184.71 DALYs per 100,000 people in 2015–2019, respectively. The economic cost of medical care doubled, being higher in Chile, but with the greatest increase in Argentina. Brazil, Peru, Argentina, and Mexico have built a better capacity for medical testing; however, typing is less frequently performed in Chile and Argentina. In 2019, Gonzalez Monsegui et al. [28] reported a higher incidence of lower respiratory infection (LRI) per 100,000 people in South American countries in Peru (9997), Bolivia (8679), and Brazil (8517). In our study, for the same year, the South American countries with the highest occurrence of influenza were Chile, Suriname, and Paraguay, with 34.34, 28.8, and 22.94 per 100,000, respectively; all together, this indicates that influenza is not the main cause of LRI morbimortality in all LAC countries.

Enzootic transmission has been established, with a rate of positivity in swine ranging from 2.0 to 36.9%; however, seropositivity has been reported to reach up to 85% in some cases [41,42,48]. Overall, among wild birds, the percentage of positive individuals was 3.32% (249 out of 7485 tests reported in the IRD), higher than that only in fowl, as reported by Hurtado et al., 2016 [40]. In poultry, the percent of positive individuals ranged from 0.7 to 12.3%, but positive individuals have reached up 30% in other studies [29,40], likely because some of them tested exclusively poultry farms with symptomatic individuals.

During outbreaks, stock losses could reach millions of animals, with an average of 282.7 (s.d. ± 1892.6) production individuals sacrificed per positive confirmed. However, losses via sacrificed animals were poorly reflected in meat export figures (Figure 2D,E), suggesting that better indicators are needed to evaluate the economic impact of these outbreaks. Most indicators are aimed to show economic growth, but Ayala and Chapa [49] used a general model that considers exports, imports, and perception to quantify the impact of H1N1 in Mexico; they conclude that this pandemic caused a decline of 1.7 per cent in the national consumption of pork meat, and losses close to USD $54 million for the Mexican market in 2009 [49]. Models like this could complement the meat price index and other international indicators that may reflect the effect of influenza on industries of the region if fully implemented. The growth of meat exports in Brazil has its roots in price regulation, industrial modernization and arrival of foreign capital, but it is also related to the management of influenza [50,51]. This country develops health programs led by Empresa Brasileira de Pesquisa Agropecuária (EMBRAPA), including biosafety protocols accepted by producers, national vaccine production, and diagnostic support; this is reflected in the score34, where Brazil appears as the only LAC country where H5N1 has been managed with post-event vaccination.

The impact of releasing an animal health alert has immediate consequences for producers and their clients. This is believed to be the main reason for the hesitance in influenza reporting that characterizes the pecuniary industries in LACs. Current requirements for importing live birds into the Andean Community include tracking the bird’s origin to areas free of influenza for at least 21 days prior to shipment, together with a lack of symptomatology, negative results in the immunodiffusion test, and not having received the AIV vaccine [52]. The worst consequences of this situation are high hesitance to implement vaccination and avoidance of animal health authorities if a sick animal is spotted.

Four cases of HPAI viruses transmitted to humans have been recorded in LAC. In one of the H5N1 cases, a 9-year-old girl presented with conjunctival pruritus and coryza, nausea, vomiting, constipation, meningitis, septic shock, and pneumonia [43,44]. The case was traced through the joint efforts of the Ecuadorian Ministry of Agriculture and Livestock and the Phytosanitary and Animal Health Regulation and Control Agency and typed as H5N1 clade 2.3.4.4b, one of the clades also infecting humans in Europe and China. The second patient, a 53-year-old man in Chile, also developed severe disease with dyspnoea and pneumonia [45]. In both cases, an epidemiological link to avian hosts was established, indicating that exposure to this variant is a real threat to human health and that its detection needs to be increased in Latin America; additionally, the frequency of H5, likely of the genotype N1, is increasing in native mammals and causes significant symptoms in those with aquatic habitats, such as sea lions and the southern river otter. From July 2023 to October 2024, HPAI viruses continued circulating in LAC. H5N1, H5N2, and H7N3 were reported in wild and domestic birds in Mexico; H5N1 is still circulating in birds in Peru, Argentina, Brazil, Colombia, Costa Rica, Ecuador, Panama, Venezuela, and Uruguay ([53], https://wahis.woah.org/#/event-management, accessed on 15 November 2024), and in sea lions in Argentina. Although no new cases of H5N1 in humans have been recorded for LAC, lineages of this serotype are being transmitted to human populations in Canada and the United States [53]. Only two cases of H5N2 in humans were recorded in FluNet. Serological data from Asian countries indicated productive infection among farm workers exposed to this genotype [54,55]; however, the infection is unlikely to generate clinical manifestations unless new mutations or re-assortment allow these variants to evolve into strains adapted to the human host.

In general, typing is partial, biased, and not at hand to take action. In the medical system, typing is centred in H1 as a consequence of the extended use of commercial panels for multiplex amplification. Data sheets in the sentinel systems have the option to register contact with dead birds and pigs; however, issues of governance prevent these records from triggering immediate actions by animal health authorities. The focus of typing in animal health changes constantly, and is presently biased to H5, but the handling of outbreaks in productive systems does not include screening among farmers. The value of typing H1 and H5 is also low because virulence to birds (HPAI phenotype) does not directly translate into pathogenicity in humans. While having contact with viruses may cause zoonotic transmission, the emergence of genotypes implies continued accumulation of genetic variation in multiple genomic segments [13,56].

In both human health and agriculture, typing for symptomatic cases is mostly performed in reference laboratories, with PCR amplification tools occasionally supplemented with amplicon sequencing. The IRD database contains data from these sequenced fragments, but most of them are disconnected from clinical indicators or epidemiology. In LAC, phylogenetic characterization is infrequent, and whether there are other differences between the same types of influenza isolated from humans and multiple animals is not usually addressed by whole genome sequencing [51]. Overall, the typing strategy generates insufficient information for the discrimination of reservoirs and the prediction of adaptations to humans. Additionally, environmental screening independent of foci of symptomatic animals is not routinely performed, reducing the tracking of LPAI and preventive measures when a HPAI combination first appears in wild populations.

The effect of climate change is shown in Supplementary Figures S3 and S4. Augments in cases in people coincide with the rainy seasons in tropical countries and the coldest weeks of winter for Mexico and areas of southern South America. Outbreaks in wildlife are becoming more intense for Mexico, central America, and the Caribbean, and tend to initiate in February rather than in March after typical post-winter migrations. Most cases of H5N1 are linked to migratory birds; these avian reservoirs usually reach LAC via three routes connecting North, Central, and South America [40], but no particular genotypes arising in South America have been tracked in birds returning from overwintering. The transmission patterns in medical networks are shifting in seasonality, and the number of human cases is becoming more similar for temperate and tropical countries. This is particularly evident for Colombia and Brazil; in them, the number of cases recorded since 2016 compares to that in Mexico and Argentina, countries with marked winter seasons and where the H1N1pdm2009 pandemic had its greatest impact (Figure 2A,B). In southern countries, there is no pattern of seasonality for the transmission of influenza between animals; other factors, likely levels of deforestation and loss of diversity, may explain the increase in AIV cases in these areas.

Data for this study were gathered from secondary sources. One of the limitations is the assumption that figures in WAHIS, IRD, and FluNet are the total of each country. In general, there is more consistency in the criteria and resources to share data for human influenza; because IRD is less popular and optional, and outbreaks are not always informed through WAHIS, the burden of influenza in animals may be underestimated. For human influenza, age-adjusted annual cases and DALYs were chosen over variables evaluated by age and sex available in primary sources, because these are easier to compare to data in animals. We focused on poultry and swine because they are the main source of meat in LAC and the principal targets of influenza surveillance for agricultural authorities; however, the analysis did not consider all their niches, for example backyard animals as compared to small producers, industrial farms, or wildlife. Similarly, the impact of influenza regarding the biogeographical range, behaviour, or population of wild species per country was considered to be outside the scope of this study.

## 5. Conclusions

The recommendations to reinforce control and surveillance of influenza in LAC, as outcomes of this study, are: (i) National public health policies for the surveillance of influenza in people should be updated according to population growth and demographic transition. Additionally, it should be noted that the sentinel system is functional, but poorly connected to surveillance mechanisms capable to foresee outbreaks. (ii) Given that surveillance in animals involves the use of time-consuming strategies for case confirmation, there is an urgent demand for new testing and typing methodologies applicable in the field. (iii) Countries should increase monitoring in wildlife by testing environmental samples in coordination with conservancy agencies, in such a way that any avian or wild mammal captured undergoes a rapid test for influenza and generates traceable data. (iv) Surveillance in poultry farms is more efficient than in swine rearing; however, for both industries, vaccination needs to be reviewed in the context of international treaties. (v) The routes of action in the event of an outbreak in animals need homogeneity and resources to extend random sampling to species different from the source of diseased animals. (vi) Finally, the implementation of One Health programs should create connection offices that generate real-time records of environmental surveillance, animal health, and human cases.

## Figures and Tables

**Table 1:** Sources of information.

Data Base	Abbreviation	Use	Link
Influenza network	FluNet	AIV and BIV cases in the medical system, per year and country	https://www.who.int/tools/flunet
Food and Agriculture Organization	FAO	Poultry and swine stocks, per year and country Poultry and pork meat exports, per year and country	https://www.fao.org/faostat/en/#data/QCL https://www.fao.org/faostat/en/#data/TI
Global Burden of Disease [32]	GBD	Disability-adjusted life years (DALYs), per year and country	https://www.healthdata.org/research-analysis/library/global-burden-369-diseases-and-injuries-1990–2019-systematic-analysis
Global Initiative on Sharing All Influenza Data	GISAID	Sources of data for influenza genomic surveillance	https://nextstrain.org/flu/
Influenza Research Database [31]	IRD	Surveillance for AIV in avian hosts and no-human mammals	https://www.bv-brc.org/searches/SurveillanceSearch
World Animal Health Information System	WAHIS	Events of avian, equine and porcine influenza tracked in productive systems	https://wahis.woah.org/#/event-management
World Bank Databank	World Bank	Population, per year and country	https://data.worldbank.org/indicator/SP.POP.TOTL?end=2022&locations=ZJ&start=2009

**Table 2:** Host range and mortality of H5N1, H5N2, H7N3 and H7N6 in LAC (2009–mid 2023).

Genotype	Host(s)	Positives Confirmed	Deaths	Sacrificed	Sources
H5(N untyped)	Coati	16	16	0	WAHIS ^1^
H5(N untyped)	Poultry	405	719	870	WAHIS ^1^
H5(N untyped)	Sea lion, fur seal, otter, Chilean dolphin, southern river otter	449	449	0	WAHIS ^1^
H5(N untyped)	Wild birds, multiple species	604	55	28	WAHIS ^1^
H5N1	Human	2	0	---	[43,44,45,46]
H5N1	Poultry	4,215,219	2,444,659	28,880,317	WAHIS
H5N1	Wild birds: includes Anseriformes, Cathartidae, Falconiadae, Galliformes, Hirundinidae and Pelecanidae	3027	2147	700	WAHIS
H5N2	Human	2	0	---	FLUNET
H5N2	Poultry	130,017	41,952	1,575,341	WAHIS
H5N2	Wild birds	2	NA	NA	IRD
H7N3	Poultry	4,562,445	1,889,150	24,482,538	WAHIS
H7N3	Wild birds, multiple species: Hirundinidae, Icteridae, Anseriformes	19	19	0	WAHIS
H7N6	Poultry	57,575	5637	442,300	[47], WAHIS
NA	Brown pelican	79	73	1	WAHIS ^1^
NA	Poultry	94,940	52,623	199,740	WAHIS ^2^

^1^ All data are symptomatic birds in the past two years, likely to be H5N1. ^2^ Argentina 2013, likely to be H5N2.

## Data Availability

Links to original data sources are provided in methods. Combined and filtered data frames are available upon request.

## Supplementary Materials

The following supporting information can be downloaded at: https://www.mdpi.com/article/10.3390/v17020225/s1, Figure S1: AIV tests performed in the poultry and swine industries 2009-2023 by animal health authorities Figure S2: Genotyping for Influenza A in LAC excluding H5N1 cases; Figure S3: Incidence of human influenza in Latin American and the Caribbean sub-regions; Figure S4: Seasons of influenza in Latin America and the Caribbean; Table S1: Direct economic cost per confirmed case of influenza; Table S2: Web pages source of vaccination data; Table S3: Detection techniques used by animal health authorities; Table S4: LAC institutions originating samples and submitting sequences for flu genomic surveillance in GSAID.
