# Supplementary material for: Disparities in Influenza Control and Surveillance in Latin America and the Caribbean
Source: Viruses. 2025 Feb 5;17(2):225. doi: 10.3390/v17020225 (PMC11861997; doi:10.3390/v17020225)
Supplement: Supplementary file 1 [file viruses-17-00225-s001.zip › viruses-3366462-supplementary.pdf]

SUPPLEMENTARY MATERIAL

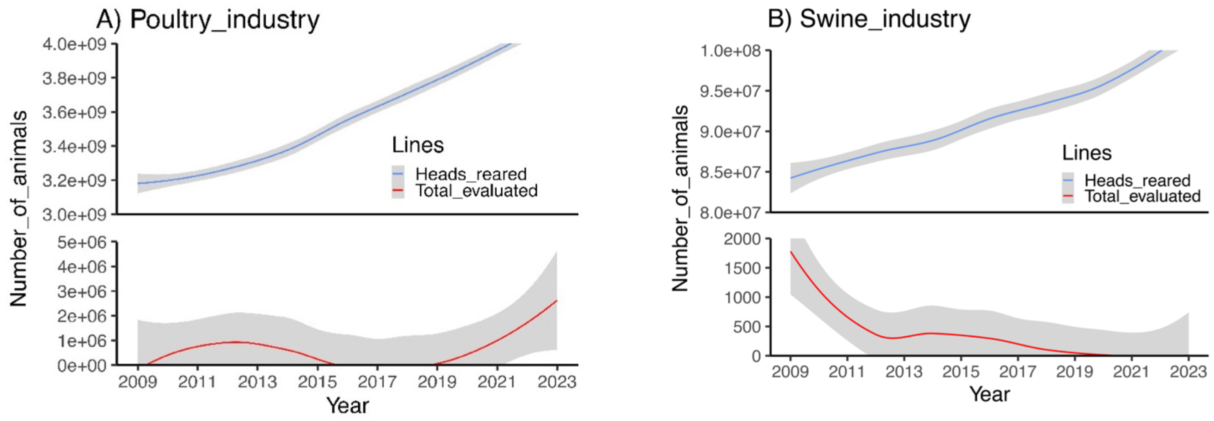

Figure S1. AIV tests performed in the poultry and swine industries 2009-2023 by animal health authorities. Data correspond to WAHIS registries, retrieved during July 2023 and plotted with 90% confidence interval.

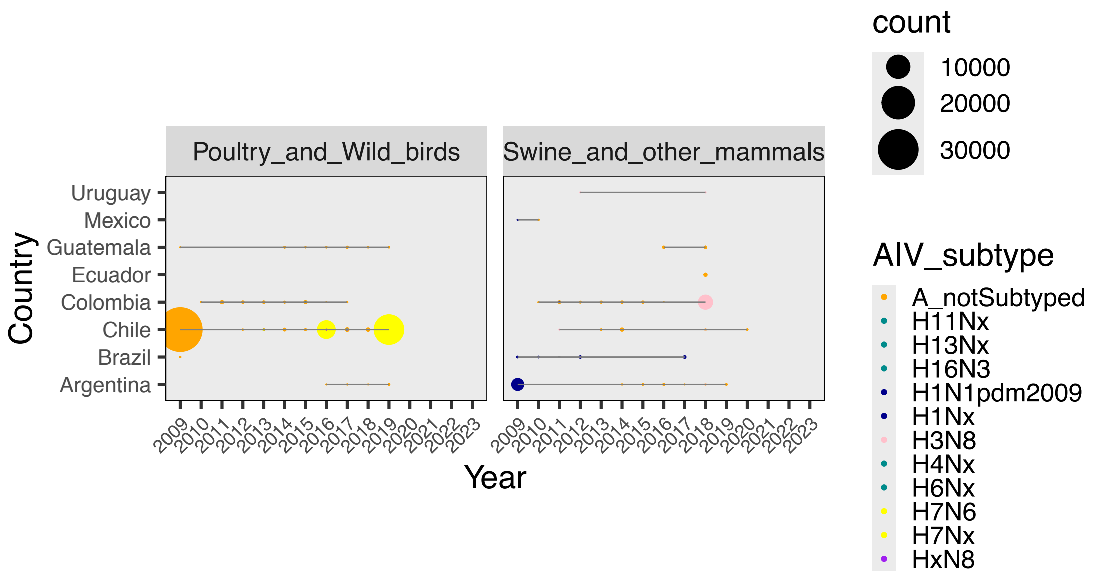

Figure S2. Genotyping for Influenza A in LAC excluding H5N1 cases. A) Coverage in avian hosts, estimated with data from IRD and WAHIS. B) Typing in non-human mammals, estimated with data from IRD and WAHIS. Data were retrieved during July 2023.

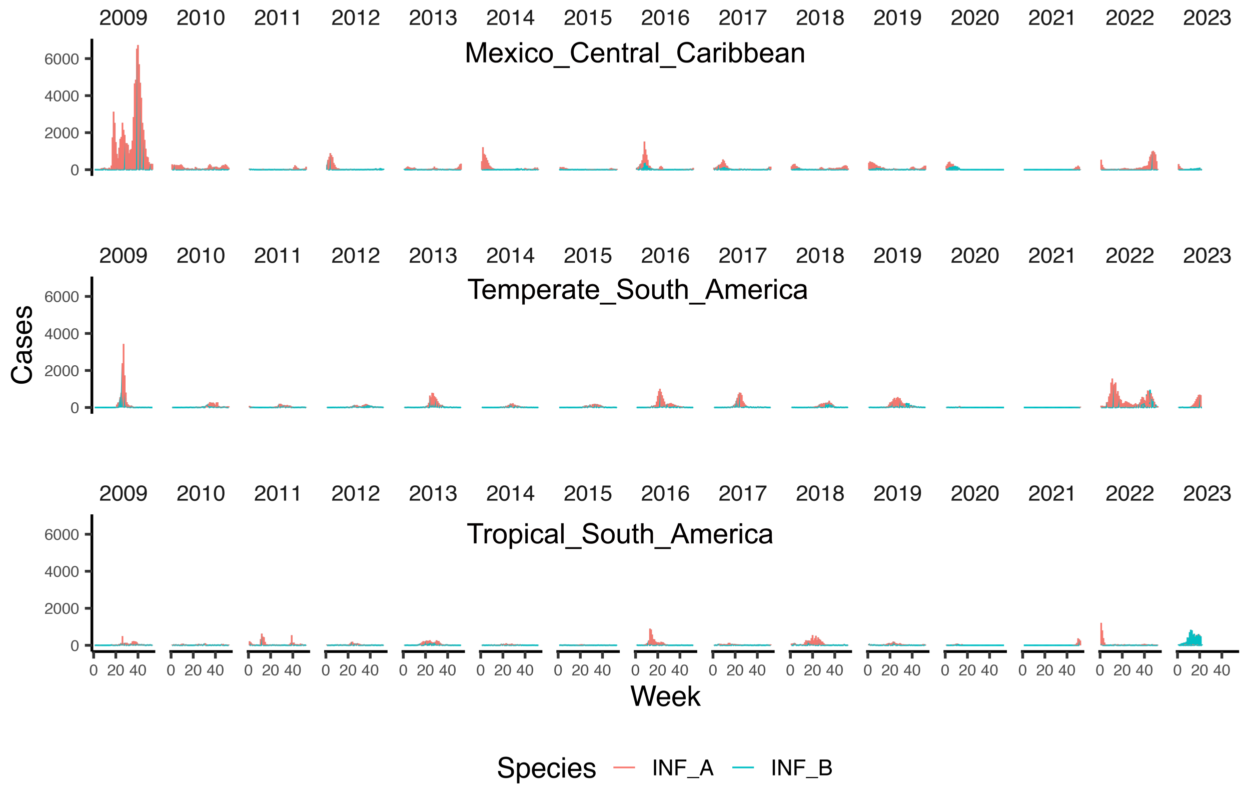

Figure S3. Incidence of human influenza in Latin American and the Caribbean sub-regions. These data only include reports in humans, some related to zoonotic outbreaks. Data were obtained from the Global Influenza Surveillance and Response System (GISRS) via the FluNet database (https://www.who.int/tools/flunet), retrieved on July 2023.

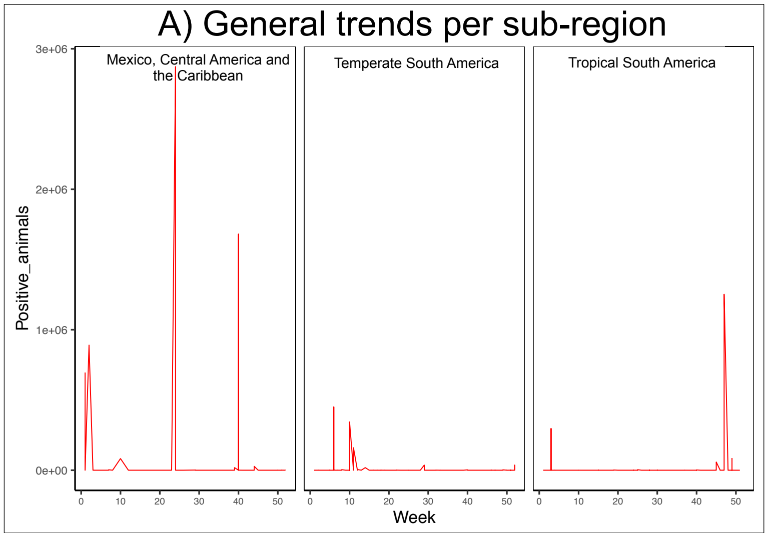

Figure S4. Seasons of influenza in Latin America and the Caribbean. A) General trends according to accumulate of historical records. Include all AIV positive animals reported by week of environmental samplings (IRD data) or by week of beginning for epidemic events reported by animal health authorities (WAHIS). B) Records per year in each sub-region. Some years (2010, 2019, 2020 and 2021) are omitted to keep the aesthetic of the plot.

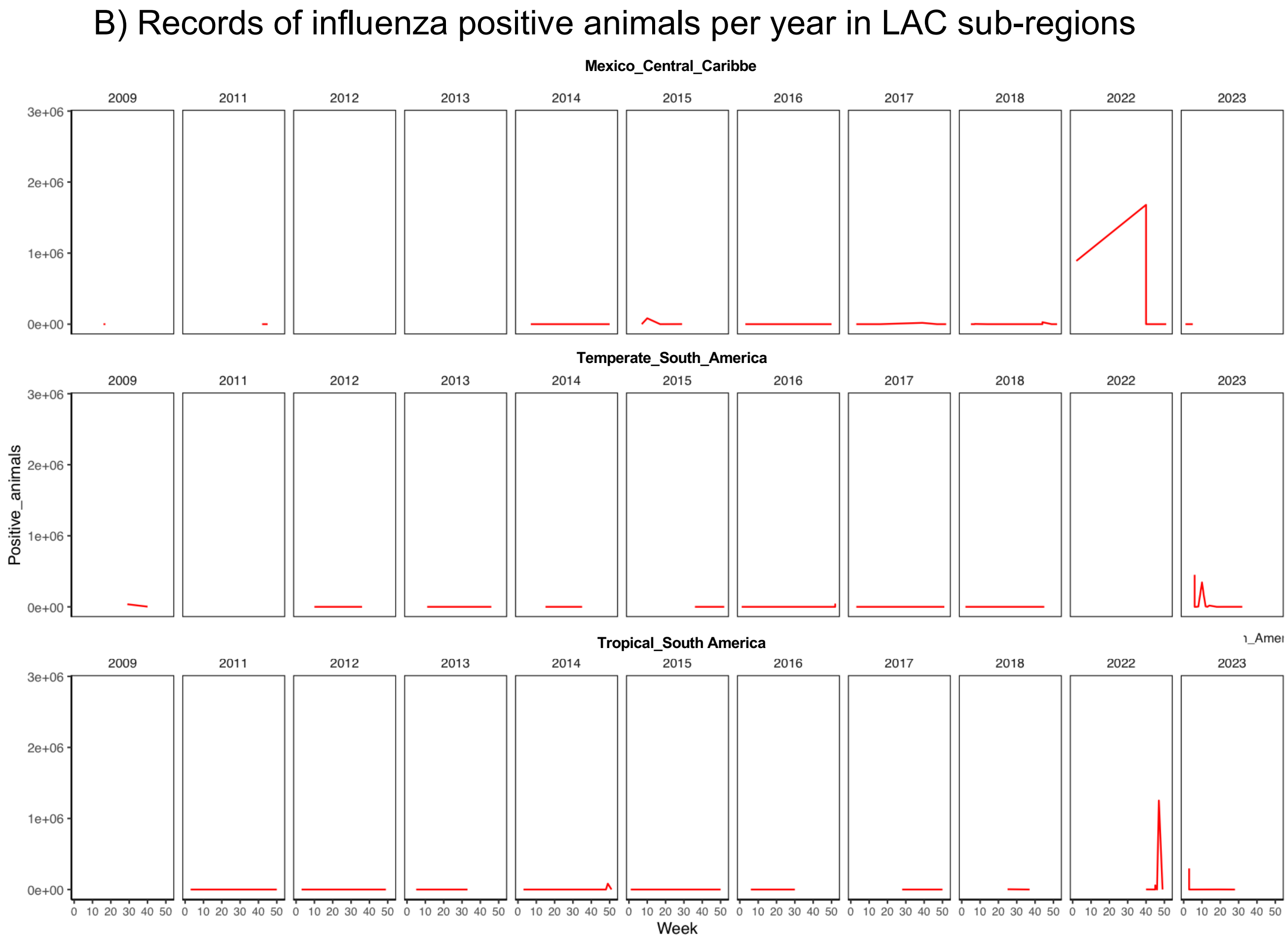

Supplementary Table S1. Direct economic cost per confirmed case of influenza.

Country	Estimated direct cost per influenza case (USD dollars), based in studies (grey cells) and adjusted by inflation rate (white cells).												Author
	2011	2012	2013	2014	2015	2016	2017	2018	2019	2020	2021	2022	
Argentina	1133	1246.3	NA	NA	NA	NA	1566.6	2103.9	3229.6	4586.0	4680.7	4745.4	Alvis-Guzmán, et al 2018
Brazil	849.5	895.4	950.9	1010.8	1101.8	1197.6	1238.3	1284.2	1331.7	1374.3	1539.9	1705.4	Alvis-Guzmán, et al 2018
Chile	4867.2	5013.2	5103.5	5343.3	5573.1	5784.9	5912.1	6048.1	6181.2	6366.6	7781.4	8452.2	Alvis-Guzmán, et al 2018
Colombia	1649.9	1702.7	1736.8	1787.1	1876.5	2017.2	2103.9	2171.3	2247.3	2303.5	2961.6	3251.9	Alvis-Guzmán, et al 2018
Colombia				1826.1	1917.4	2061.2	2149.8	2218.6	2296.3	2353.7	3026.2	3322.9	Salcedo-Mejia et al. 2019
Colombia								847.0	1089.0	1116.2	1435.1	1575.8	Castillo-Rodriguez et al. 2022
Guatemala	524.8	544.7	568.2	587.5	601.6	628.1	655.7	680.6	705.8	728.4	897.8	1027.9	Alvis-Guzmán, et al 2018
Honduras	722.8	760.4	799.9	848.7	875.9	899.5	934.6	974.8	1017.7	1053.3	1287.4	1428.8	Alvis-Guzmán, et al 2018
Nicaragua	314.9	337.6	361.5	383.2	398.6	412.5	428.6	449.6	473.9	491.4	591.7	648.1	Alvis-Guzmán, et al 2018
Uruguay	2201.8	2380.1	2584.8	2814.9	3059.8	3353.5	3561.4	3832.1	4134.8	4540.1	5129.7	5693.4	Alvis-Guzmán, et al 2018

Supplementary Table S2. Web pages source of vaccination data.

Country	Dependence	Vaccine strain	Web site
Argentina	Dirección Nacional de Sanidad Animal del SENASA	H5N1	www.senasa.gob.ar
Bolivia	Servicio Nacional de Sanidad Agropecuaria e Inocuidad Alimentaria (SENASAG)	H5N1	www.senasag.gob.bo
Brazil	Ministerio da Agricultura, Pecuária e Abastecimento	H5N1	www.agricultura.gov.br
Chile	Servicio Agrícola y Ganadero (SAG)	No data available	www.sag.cl
Colombia	Instituto Colombiano Agropecuario	H5N1	www.ica.gov.co
Costa Rica	Ministerio de Salud	No data available	www.ministeriodesalud.go.cr
Cuba	Ministerio de Agricultura	No details	www.presidencia.gob.cu
Ecuador	Ministerio de Agricultura y Ganadería	No vaccination	www.agrocalidad.gob.ec
El Salvador	Ministerio de Agricultura y Ganadería	H5	www.mag.gob.sv
Guatemala	Ministerio de Agricultura, Ganadería y Alimentación	No details	www.visar.maga.gob.gt
Honduras	Secretaría de Agricultura y Ganadería	No details	www.sag.gob.hn

Supplementary Table S3. Detection techniques used by animal health authorities

Country	Year	Detection_techniques	Source
Argentina	2009	Viral isolation, qRT-PCR, sequencing	WAHIS
Brazil	2009	qRT-PCR, Sanger sequencing	Hurtado et al. 2016
Brazil	2009	Nested RT-PCR, Sanger sequencing	Rajão et al. 2013
Chile	2009	Viral isolation, NIA, HIT, Ab-ELISA, qRT-PCR, Sanger sequencing	WAHIS
Mexico	2009	Virus isolation, RT-PCR, qRT-PCR, Sequencing	WAHIS
Brazil	2010	HI-test, Sanger sequencing, qRT-PCR	Ciacci-Zanella, et al. 2015; Schaefer et al. 2015
Brazil	2011	HI-test, Sanger sequencing, qRT-PCR	Ciacci-Zanella, et al. 2015; Nelson et al. 2015
Chile	2011	Viral isolation, qRT-PCR	WAHIS
Brazil	2012	Nested RT-PCR, Sanger sequencing	Fraiha et al. 2021, Haach et al. 2020
Mexico	2012	Viral isolation, NIA, IVPI	WAHIS
Uruguay	2012	qRT-PCR, Sanger sequencing	WAHIS
Mexico	2013	Viral isolation	WAHIS
Belize	2014	Viral isolation, AGI, HI test, qRT-PCR	WAHIS
Mexico	2014	NA	WAHIS
Mexico	2015	Viral isolation, IVPI	WAHIS
Mexico	2015	Viral isolation, IVPI	WAHIS
Mexico	2015	IVPI	WAHIS
Chile	2016	Ab-ELISA, qRT-PCR	Jimenez-Bluhm et al. 2019
Chile	2016	AGI, NIA, RT-PCR, qRT-PCR, Sequencing	WAHIS
Brazil	2017	Nested RT-PCR, Sanger sequencing	Fraiha et al. 2021, Haach et al. 2020
Dominican_F	2017	HIT, AGI, RT-PCR, qRT-PCR, Sequencing	WAHIS
Mexico	2017	Viral isolation, IVPI, Sequencing, qRT-PCR	WAHIS
Argentina	2018	qRT-PCR	WAHIS
Chile	2018	qRT-PCR	WAHIS
Colombia	2018	AGI, HIT, qRT-PCR	WAHIS
Dominican_F	2018	[illegible] Sequencing	WAHIS
Ecuador	2018	ELISA, qRT-PCR	WAHIS
Mexico	2018	Viral isolation, NIA, HIT, IVPI, Sequencing	WAHIS
Uruguay	2018	qRT-PCR	WAHIS
Chile	2019	qRT-PCR, Sequencing	WAHIS
Mexico	2019	Viral isolation, IVPI, Sequencing	WAHIS
Chile	2022	NGS sequencing	WAHIS
Colombia	2022	qRT-PCR	WAHIS
Ecuador	2022	RT-PCR, NGS sequencing	WAHIS
Ecuador	2022	qRT-PCR, sequencing	WAHIS
Honduras	2022	qRT-PCR	WAHIS
Mexico	2022	IVPI, Viral isolation, qRT-PCR, Sequencing	WAHIS
Mexico	2022	Inoculation in chicken embryo, qRT-PCR	WAHIS
Mexico	2022	Virus isolation, qRT-PCR, Sequencing	WAHIS
Mexico	2022	Virus isolation, qRT-PCR, Sequencing	WAHIS
Panama	2022	qRT-PCR	WAHIS
Peru	2022	qRT-PCR	WAHIS
Peru	2022	RT-PCR	WAHIS
Venezuela	2022	RT-PCR	WAHIS
Ecuador	2022	qRT-PCR, WGS sequencing	EFSA et al. 2023
Argentina	2023	qRT-PCR	WAHIS
Argentina	2023	qRT-PCR	WAHIS
Argentina	2023	qRT-PCR	WAHIS
Bolivia	2023	RT-PCR mutiplex, qRT-PCR, sequencing	WAHIS
Bolivia	2023	Multiplex RT-PCR, qRT-PCR	WAHIS
Brazil	2023	qRT-PCR, NGS sequencing	WAHIS
Chile	2023	AGI, qRT-PCR, NGS sequencing	WAHIS
Chile	2023	qRT-PCR, NGS sequencing	WAHIS
Chile	2023	AGI, qRT-PCR, NGS sequencing	WAHIS
Chile	2023	AGI, qRT-PCR, NGS sequencing	WAHIS
Chile	2023	AGI, RT-PCR, qRT-PCR, NGS sequencing	WAHIS
Chile	2023	AGI, qRT-PCR, NGS sequencing	WAHIS
Chile	2023	qRT-PCR	WAHIS
Chile	2023	qRT-PCR	PAHO
Colombia	2023	qRT-PCR	WAHIS
Costa Rica	2023	qRT-PCR, RT-PCR, NGS sequencing	WAHIS
Cuba	2023	qRT-PCR	WAHIS
Guatemala	2023	qRT-PCR	WAHIS
Paraguay	2023	RT-PCR	WAHIS
Peru	2023	qRT-PCR, WGS sequencing	WAHIS
Uruguay	2023	qRT-PCR	WAHIS
Uruguay	2023	qRT-PCR, WGS sequencing	WAHIS
Uruguay	2023	qRT-PCR	WAHIS

Ab-ELISA= Antibody-detection ELISA, AGI=Agar-gel immunodiffusion, HIT=Hemagglutination inhibition test, IVPI=Intravenous pathogenicity index, NIA=Neuraminidase inhibition assay

Supplementary Table S4. LAC institutions originating samples and submitting sequences for flu genomic surveillance in GSAID. (Those that have been generating sequencing capacity and now carry out both processes are highlighted in green).

Country	Originating_labs	Submitting_labs	Surveillance_
Argentina	Instituto Nacional de Enfermedades Infecciosas INEI-ANLIS Carlos G. Malbran	Centers for Disease Control and Prevention	seasonal
	Instituto Nacional de Enfermedades Infecciosas INEI-ANLIS Carlos G. Malbran	Crick Worldwide Influenza Centre	seasonal
	Instituto Nacional de Enfermedades Infecciosas INEI-ANLIS Carlos G. Malbran	Instituto Nacional de Enfermedades Infecciosas INEI-ANLIS Carlos G. Malbran	seasonal
	National Institute for Medical Research	Instituto Nacional de Enfermedades Infecciosas INEI-ANLIS Carlos G. Malbran	seasonal
Aruba	National Institute for Public Health and the Environment, RIVM	National Institute for Public Health and the Environment, RIVM	seasonal
Bolivia	Instituto Nacional De Laboratorios de Salud, INLASA	Centers for Disease Control and Prevention	seasonal
	Centro Nacional de Enfermedades Tropicales, CENETROP	Centers for Disease Control and Prevention	seasonal
Brazil	Evandro Chagas Institute	Evandro Chagas Institute	seasonal
	Laboratorio Central de Saúde Pública de Alagoas	Oswaldo Cruz Foundation	seasonal
	Laboratório Central do Estado do Paraná	Instituto Oswaldo Cruz Fiocruz Laboratory of Respiratory Viruses and Measles, LVRS	seasonal
	Laboratório Central De Saúde Pública Professor Gonçalo Moniz	Instituto Oswaldo Cruz Fiocruz Laboratory of Respiratory Viruses and Measles, LVRS	seasonal
	Instituto Adolfo Lutz National Influenza Center	Centers for Disease Control and Prevention	seasonal
	Laboratório Central de Saúde Pública do Distrito Federal	Laboratório Central de Saúde Pública do Distrito Federal	seasonal
	Laboratório Central de Saúde Pública do Espírito Santo	Instituto Oswaldo Cruz Fiocruz Laboratory of Respiratory Viruses and Measles, LVRS	seasonal
	Instituto Adolfo Lutz National Influenza Center	Instituto Adolfo Lutz National Influenza Center	seasonal
	Fundacao Oswaldo Cruz Mato Grosso do Sul	Instituto Oswaldo Cruz Fiocruz Laboratory of Respiratory Viruses and Measles, LVRS	seasonal
	Instituto Oswaldo Cruz Fiocruz Laboratory of Respiratory Viruses and Measles, LVRS	Centers for Disease Control and Prevention	seasonal
	Ezequiel Dias Foundation, FUNED	Centers for Disease Control and Prevention	seasonal
	Laboratório Central de Saúde Pública da Paraíba	Evandro Chagas Institute	seasonal
	Laboratório Central do Estado do Paraná	Instituto Oswaldo Cruz Fiocruz Laboratory of Respiratory Viruses and Measles, LVRS	seasonal
	Laboratório Central de Saúde Pública de Pernambuco	Evandro Chagas Institute	seasonal
	Laboratório Central de Saúde Pública do Rio de Janeiro	Instituto Oswaldo Cruz Fiocruz Laboratory of Respiratory Viruses and Measles, LVRS	seasonal
	Instituto Oswaldo Cruz Fiocruz Laboratory of Respiratory Viruses and Measles, LVRS	Instituto Oswaldo Cruz Fiocruz Laboratory of Respiratory Viruses and Measles, LVRS	seasonal
	Laboratório Central de Saúde Pública do Rio Grande do Sul	Instituto Oswaldo Cruz Fiocruz Laboratory of Respiratory Viruses and Measles, LVRS	seasonal
	Laboratorio Central de Saúde Pública de Alagoas	Instituto Oswaldo Cruz Fiocruz Laboratory of Respiratory Viruses and Measles, LVRS	seasonal
	Instituto de Biotecnologia, IBTEC	Instituto de Biotecnologia, IBTEC	seasonal
	University of Sao Paulo	Oswaldo Cruz Foundation	seasonal
	Instituto Butantan	Instituto Butantan	seasonal
	Instituto Adolfo Lutz National Influenza Center	Instituto Adolfo Lutz National Influenza Center	seasonal
	Instituto Adolfo Lutz Regional De Santo Andre	Instituto Adolfo Lutz National Influenza Center	seasonal
	Laboratório Central de Saúde Pública de Sergipe Instituto Parreira Horta	Laboratório Central de Saúde Pública de Sergipe Instituto Parreira Horta	seasonal
	Ezequiel Dias Foundation, FUNED	Ezequiel Dias Foundation, FUNED	seasonal
	Laboratório Central de Saúde Pública do Amazonas	Evandro Chagas Institute	seasonal
	Instituto Adolfo Lutz National Influenza Center	Evandro Chagas Institute	seasonal
	Laboratório Central de Saúde Pública do Rio Grande do Sul	Laboratório Central de Saúde Pública do Rio Grande do Sul	seasonal
	University of Sao Paulo	Instituto Oswaldo Cruz Fiocruz Laboratory of Respiratory Viruses and Measles, LVRS	seasonal
	Laboratório Central de Saúde Pública de Sergipe Instituto Parreira Horta	Instituto Oswaldo Cruz Fiocruz Laboratory of Respiratory Viruses and Measles, LVRS	seasonal
Chile	Instituto de Salud Pública de Chile	Instituto de Salud Pública de Chile	seasonal
	Instituto de Salud Pública de Chile	Centers for Disease Control and Prevention	seasonal
Colombia	Caribbean Epidemiology Center	Centers for Disease Control and Prevention	seasonal
	Instituto Nacional de Salud de Colombia	Instituto Nacional de Salud de Colombia	seasonal
	Instituto Nacional de Salud de Colombia	Centers for Disease Control and Prevention	seasonal
CostaRica	Sección de Diagnóstico Molecular, Hospital Clínica Bíblica	Inciensa, Investigación y Enseñanza en Nutrición y Salud	seasonal
	Inciensa, Investigación y Enseñanza en Nutrición y Salud	Inciensa, Investigación y Enseñanza en Nutrición y Salud	seasonal
DomRep	Laboratorio Nacional de Salud Pública Dr. Defillo	Laboratorio Nacional de Salud Pública Dr. Defillo	seasonal
Ecuador	Instituto Nacional de Investigación en Salud Pública, INSPI	Centers for Disease Control and Prevention	seasonal
ElSalvador	Laboratorio Central Dr. Max Bloch, contiguo Hospital Rosales	Centers For Disease Control And Prevention	seasonal
	CNR Influenza France Sud	Crick Worldwide Influenza Centre	seasonal

French_terr	Erasmus Medical Center	Erasmus Medical Center	seasonal
	CNR Influenza France Sud	Centre National de Reference	seasonal
	CNR Virus des Infections Respiratoires France Sud	Center For Infectious Diseases Research	seasonal
	CNR Influenza France Sud	National Institute For Medical Research	seasonal
	Institut Pasteur	Crick Worldwide Influenza Centre	seasonal
	Institut Pasteur	National Institute for Medical Research	seasonal
	National Influenza Center French Guiana and French Indies	Centers for Disease Control and Prevention	seasonal
	Institut Pasteur	Institut Pasteur	seasonal
	National Influenza Center French Guiana and French Indies	Who Chinese National Influenza Center	seasonal
Guatemala	Laboratorio Nacional De Salud, Guatemala	Centers For Disease Control And Prevention	seasonal
	Laboratorio Nacional de Salud, Guatemala	Laboratorio Nacional De Salud, Guatemala	seasonal
Haiti	Laboratoire National de Sante Publique	Centers for Disease Control and Prevention	seasonal
Honduras	Laboratorio Nacional de Virologia	Centers for Disease Control and Prevention	seasonal
Mexico	Centro De Investigaciones Regionales Dr. Hideyo Noguchi	Universidad Autonoma De Yucatan	seasonal
	Universidad Autonoma de Yucatan	Universidad Autonoma De Yucatan	seasonal
	Laboratorio de Virus Respiratorios	Centers for Disease Control and Prevention	seasonal
Nicaragua	St. Jude Children's Research Hospital	St. Jude Children's Research Hospital	seasonal
Panama	Instituto Conmemorativo Gorgas de Estudios de la Salud	Centers for Disease Control and Prevention	seasonal
Paraguay	Laboratorio Central de Salud Pública	Centers for Disease Control and Prevention	seasonal
Peru	US Naval Medical Research Unit 6, NAMRU 6	Centers for Disease Control and Prevention	seasonal
	Laboratorio de Referencia Nacional Virus Respiratorios, Instituto Nacional de Salud	Centers for Disease Control and Prevention	seasonal
	Center for Veterinary Diagnostics Regional Animal Health Office No.6	CNR Virus des Infections Respiratoires, France Sud	seasonal
	Instituto de Medicina Tropical Alexander von Humboldt Universidad Peruana Cayetano Heredia	Instituto de Medicina Tropical Alexander von Humboldt Universidad Peruana Cayetano Heredia	seasonal
	US Naval Medical Research Unit 6, NAMRU 6	US Naval Medical Research Unit 6, NAMRU 6	seasonal
	Queen Elizabeth Hospital Department of Laboratory Medicine	Public Health Agency of Canada, PHAC	seasonal
Puerto_Rico	US Air Force School Of Aerospace Medicine	US Air Force School of Aerospace Medicine	seasonal
Suriname	Institute for Biomedical Sciences	Centers for Disease Control and Prevention	seasonal
	Central Laboratory, Bureau of Public Health	Centers for Disease Control and Prevention	seasonal
Uruguay	Departamento de Laboratorio de Salud Pública, DLSP	Centers for Disease Control and Prevention	seasonal
	Departamento de Laboratorio de Salud Pública, DLSP	Departamento de Laboratorio de Salud Pública, DLSP	seasonal
Venezuela	Instituto Nacional de Higiene Rafael Rangel	Centers for Disease Control and Prevention	seasonal
Puerto_Rico	Puerto Rico Department of Health	Centers for Disease Control and Prevention	seasonal
Chile	Instituto de Salud Pública de Chile	Instituto de Salud Pública de Chile	avian
Colombia	Instituto Colombiano Agropecuario, ICA	Instituto Nacional de Salud de Colombia	avian
CostaRica	Inciensa, Investigación y Enseñanza en Nutrición y Salud	Servicio Nacional de Salud Animal, SENASA	avian
	Servicio Nacional de Salud Animal, SENASA	Inciensa, Investigación y Enseñanza en Nutrición y Salud	avian
Ecuador	Instituto Nacional de Investigación en Salud Pública, INSPI	Instituto Nacional de Investigación en Salud Pública, INSPI	avian
	Agrocalidad	Instituto Nacional de Investigación en Salud Pública, INSPI	avian
	Ministerio del Ambiente, Agua y Transición Ecológica, MAATE	Instituto Nacional de Investigación en Salud Pública, INSPI	avian
Honduras	Instituto Hondureño de Investigaciones Medico Veterinarias SAG- Senasa	National Veterinary Services Laboratories USDA	avian
Mexico	Universidad Nacional Autónoma de México	Universidad Nacional Autonoma De Mexico	avian
Panama	Laboratorio de Diagnóstico de Enfermedades Vesiculares, LADIVES	National Veterinary Services Laboratories USDA	avian
Peru	Servicio Nacional de Sanidad Agraria del Perú, SENASA	National Veterinary Services Laboratories, USDA	avian
	Pontificia Universidad Católica del Peru	Servicio Nacional Forestal y de Fauna Silvestre, SERFOR	avian
	Universidad Privada San Juan Bautista	Farmacológicos Veterinarios SAC, FARVET S.A.C	avian
	Laboratorio de Virus Respiratorios, Centro Nacional de Salud Pública	Instituto Nacional de Salud Perú	avian
	Instituto Nacional De Salud Peru	Instituto Nacional de Salud Perú	avian
Venezuela	Instituto Nacional de Salud Agrícola Integral	Instituto Venezolano de Investigaciones Científicas	avian
	Instituto Venezolano de Investigaciones Científicas	Instituto Nacional de Higiene Rafael Rangel	avian
